# Dynamic data visualizations as events: effects of framing and change salience on segmenting dynamic maps

**DOI:** 10.1186/s41235-025-00678-7

**Published:** 2025-10-30

**Authors:** Reena Pauly, Stephan Schwan

**Affiliations:** Leibniz-Institut für Wissensmedien, Tübingen, Germany

**Keywords:** Event segmentation, Data visualization, Animation, Dynamic maps, Spatiotemporal patterns

## Abstract

Event segmentation theory, which explores how individuals divide continuous experiences into discrete events, has been extensively studied in naturalistic stimuli. We investigate whether key findings generalize to animated data visualizations, specifically dynamic thematic maps. Experiment 1 showed that inter-individual segmentation agreement in dynamic maps occurs above chance levels and is influenced by the direction of the depicted trend. Experiments 2 and 3 build on these findings by systematically varying the depicted trend in maps showing population changes of fictional insect species. In addition, we examined how conceptual (framing of the species as endangered or invasive) and perceptual factors (salience of directional change) interact to shape segmentation agreement. In Experiment 2, salience was manipulated using different color scales: Saturation-based scales as the high-salience condition and hue-based scales as the low-salience condition. We found a significant three-way interaction between trend, framing, and salience: Agreement was higher when the framing matched the trend direction, but only in the high-salience condition. In Experiment 3, salience was more subtly manipulated by showing the trend either spatially clustered (high salience) or spatially distributed (low salience) across the maps. The results partly replicate the findings of Experiment 2, showing a significant interaction between trend, framing, and spatial pattern on segmentation agreement, with higher agreement for negative trends when population decline was salient and framed as endangered. These findings suggest that symbolic visualizations are subject to event segmentation processes, provided both bottom-up perceptual features and top-down conceptual expectations support the formation and updating of internal event models.

## Significance statement

Dynamic maps are a powerful way to visualize how different variables change across time and space. They are increasingly used to communicate complex and pressing issues, such as the spread of infectious diseases, the effects of climate change, shifts in biodiversity, or spatial patterns in voting behavior. These visualizations help make large-scale trends visible and understandable to the general public. However, they are often dense and complex, which may limit their effectiveness. To improve how dynamic maps are used and understood, we need to better understand how people perceive and interpret them. In this study, we found that viewers naturally segment dynamic maps into meaningful events, much like how we divide stories or films into distinct scenes. Importantly, viewers showed substantial agreement in how they segmented the same maps—especially when the changes aligned with their expectations and were made more noticeable through clear color scales or clustered spatial patterns. These findings offer insights into how people cognitively process complex visual information and how dynamic data visualizations can be designed more effectively. Prior research shows that how people segment unfolding events affects what they remember. By extending this understanding to animated maps, our results can inform design recommendations grounded in cognitive theory and empirical evidence—supporting clearer communication of important data in science, policy, and education.

## Introduction

To better comprehend our changing world, the collection, visualization, and publication of spatiotemporal data are at an all-time high. Understanding how people perceive, process, and mentally represent these visualizations is relevant for these efforts to be fruitful. While various visualization formats exist, thematic maps are often chosen for their ability to depict properties of geographical space that are not directly observable due to their nature or the spatial and temporal scale (Hegarty, 2011). Dynamic thematic maps show how those properties evolve over time. They can help to understand and communicate a wide range of phenomena, such as voting behavior, global warming, or threats from infectious diseases (Lloyd et al., 2019). Increasing polarity and dispute over such topics underscore the need to study the fundamental perceptual and cognitive processes involved in the interplay of the visual communication of important data and their understanding by the audience.

From a cognitive perspective, visuospatial displays decrease the demand for memory resources. They organize information, allowing cognitive processes to be replaced by perceptual ones (Hegarty, 2011). Dynamic thematic maps could, therefore, be a powerful way to communicate complex spatiotemporal data patterns. However, due to the dense information presentation, recognizing those patterns is not always effective (Cybulski, 2022). Efforts to optimize the animation design to lessen the cognitive load are restricted by the nature of the temporal, geographic data themselves (Fish, 2015): States and regional borders do not necessarily conform to the limits of working memory—requiring the use of visual chunking strategies by the viewer (Stieff et al., 2020). Research has further shown that animating learning content does not necessarily facilitate learning per se but can be overwhelming or distracting if not designed well (Tversky et al., 2002). However, in a meta-analysis, Berney and Bétrancourt (2016) found an overall advantage of animated over static learning material. These advantages of animation were especially relevant in the absence of accompanying text and for the depiction of natural phenomena. Therefore, these results support the use of animated, and hence dynamic, maps for the effective visualization of spatiotemporal data.

An enhanced underpinning of the usability of dynamic maps through cognitive theories and empirical findings has also been posed as an important research objective by the cartographic research community (Battersby and Goldsberry, 2010; Montello, 2002; MacEachren and Kraak, M.- J., 2001; Fish, 2015). The research presented here strives to add to that underpinning by investigating how people mentally structure dynamic maps during observation. This structuring has to be considered as an early and important process in understanding, assuming that the way information is structured during perception also influences how it is remembered and how it can influence previously held beliefs and attitudes. Event segmentation is a well-researched structuring mechanism in human perception (Zacks, 2004; Kurby and Zacks, 2008; Zacks, 2020).

Event segmentation, as described by Zacks (2020), involves discretizing the continuous stream of perceptual input into event units, easing further processing. It has been found to be an automatic process that influences higher cognition, such as memory and learning (Zacks and Swallow, 2007). fMRI research has shown that shifts in brain activity align with perceived event boundaries, supporting the idea that event segmentation is reflected in hierarchical neural dynamics that organize continuous experience into discrete, memory-relevant units (Baldassano et al., 2017; Zacks and Swallow, 2007; Kurby and Zacks, 2008). One common behavioral paradigm to assess when event boundaries are perceived is the segmentation task, in which participants identify boundaries between meaningful units in a continuous stimulus (Newtson, 1973; Zacks, 2020). Empirical studies of event segmentation typically have participants watch and segment on videos of actors performing everyday actions such as preparing breakfast or decorating a room (Flores et al., 2017; Sargent et al., 2013), but movie scenes (Sasmita and Swallow, 2022), audio dramas (Papenmeier et al., 2019), a live football match (Huff et al., 2017) as well as basketball clips (Newberry and Bailey, 2019; Feller et al., 2023) have also been employed. According to event segmentation theory (EST), segmentation reflects the updating of event models, working memory representations that maintain predictions about ongoing activity (Zacks and Swallow, 2007; Radvansky and Zacks, 2011). Event models maintain information about the current situation and guide comprehension, prediction, and memory encoding. Transient increases in prediction errors prompt an update to the current event model, leading to the perception of an event boundary (Flores et al., 2017; Kurby and Zacks, 2008; Kumar et al., 2023).

The studied stimuli have in common that observers can be assumed to have some notion of how such scenes characteristically evolve, scripts or schemata, which is postulated to influence their segmentation behavior (Kurby and Zacks, 2008). Both perceptual and conceptual factors have been shown to guide event segmentation. Visual change can trigger the perception of event boundaries even in the absence of rich semantic context. For example, Zacks (2004) used animations of simple geometric shapes to demonstrate that changes in motion parameters (e.g., speed, direction) influenced placement of event boundaries. Moreover, their results show that the influence of the motion parameters was moderated by top-down interpretations of the unfolding scene: intentions inferred by the participants could shift where boundaries were placed. Similar results from naturalistic stimuli suggest that the impact of perceptual changes is shaped by conceptual expectations (Cutting et al., 2012; Kurby and Zacks, 2008). Domain experts, for example, show higher agreement when segmenting scenes from their area of expertise than when segmenting scenes they are less familiar with (Newberry et al., 2021).

Observers of dynamic maps can often not be expected to have elaborate event schemata or prior knowledge to build upon, eliciting the question of whether event segmentation plays a relevant role in the processing of such symbolic perceptual input. Evidence from research with abstract and simplified stimuli provides an entry point to this question. Segmentation has also been studied with abstract stimuli. As introduced above, Zacks (2004) showed that even highly simplified animations can elicit reliable event boundaries, and that both perceptual change and top-down interpretation influence where those boundaries are placed. Hard et al. (2006) similarly used animated geometric figures to demonstrate that perceiving event structure is fundamental to building event schemas, rather than being solely dependent on preexisting knowledge of intentions. Together, these findings indicate that segmentation can occur in the absence of complex semantic content or naturalistic motion. This is relevant when considering symbolic displays such as animated thematic maps.

Animated choropleth maps possess unique characteristics that distinguish them from other moving visual stimuli, which might shape how viewers perceive and segment events. For one, animated choropleth maps do not offer any direct movement features, as the depicted objects—regions of the map—remain stationary. The only feature that changes over time is the color. Although the depicted regions remain spatially stationary, temporal changes in their color values, especially those involving shifts in luminance, can elicit low-level motion signals (Mather, 1984), which in turn might trigger segmentation. Furthermore, maps usually do not show easily identifiable actors—be they humans or geometric shapes—whose goals could be inferred, as they depict spatially aggregated data. Another aspect to consider when discussing the segmentation of dynamic maps, which is, in essence, a temporal structuring mechanism, is the special relationship between event duration and observation duration. Thematic maps are especially useful for communicating phenomena that develop on a spatial or temporal scale that are not directly perceivable by humans. While observations in everyday life occur in real-time, a dynamic map may display 10 days or 10 centuries in the view time of a minute.

In summary, dynamic maps differ from traditional stimuli both in the perceptual (bottom-up) and conceptual (top-down) attributes which have been shown to guide segmentation (Zacks, 2004; Hard et al., 2006; Kurby and Zacks, 2008; Zacks, 2020). Perceptually, they lack continuous object motion and rely solely on color changes in fixed spatial units. Conceptually, they often depict abstract, aggregated data without actors or familiar scripts that could anchor event models. Prior work has already demonstrated that segmentation can occur with abstract animations of simple figures (Zacks, 2004; Hard et al., 2006), where both perceptual change and inferred intentions play a role. The present study builds on this work by examining whether event segmentation similarly occurs in the highly symbolic and temporally compressed context of animated choropleth maps—stimuli that provide minimal perceptual motion cues and little conceptual context in the form of familiar scripts or actors. Such a finding would support the view that event segmentation is a general perceptual–cognitive process, not dependent on object motion or well-developed event schemas.

### The current study

This study aims to achieve two objectives by applying an event segmentation paradigm to dynamic maps: First, as a theoretical contribution, it tests whether the differentiation of conceptual and perceptual influences on segmentation generalizes to abstract stimuli such as data visualizations. Second, it empirically investigates the inter-individually shared processing of data depicted on dynamic maps.

We assess natural segmentation, neither instructing a coarse nor fine-grained segmentation (Jafarpour et al., 2022). Explicitly, participants were instructed to watch animated maps and press the space bar whenever they perceived that one meaningful event had ended and another meaningful event had begun. It was stressed that there were no right or wrong times to press the spacebar key.

As the outcome measure, this research focuses on intersubjective segmentation agreement (Zacks et al., 2001), defined in detail in the Design section of Experiment 1. This focus is motivated by the need to understand consistency in how individuals perceive and segment dynamic maps to be able to derive design implications in the future that can be assumed to benefit the majority of the map users. Put differently, this study aims to explore the shared cognitive understanding as a preliminary to be able to tackle the question of which characteristics of maps and viewers lead to variances in that understanding.

To that end, we present three experiments: In the first study (Section Experiment 1: Segmentation Agreement for Dynamic Maps), we test the applicability of the segmentation paradigm to assess the inter-individually shared processing of dynamic thematic map stimuli. In the two following experiments (Section Experiment 2: Framing and Color Scale and Experiment 3: Framing and Spatial Pattern ), we independently manipulated the conceptual interpretation of the depicted data trends and the perceptual salience of the direction of those trends to differentiate top-down and bottom-up influences on segmentation. The pre-registrations for the three reported experiments can be found here: https://osf.io/v9n3m/overview.

Based on reviewer feedback, additional analyses focusing on segmentation frequency have been added for a more complete investigation of the effect of the experimental conditions on the segmentation process. These are marked as exploratory, as they were not preregistered.

## Experiment 1: Segmentation agreement for dynamic maps

As discussed in the introduction, dynamic thematic maps differ from the stimuli previously studied in the event segmentation research on several relevant dimensions: movement, identifiable agents, the relationship between event and observation duration, and the availability of scripts and schemata (Zacks, 2020). Therefore, the primary research question of the first experiment was whether event segmentation is a relevant process in the perception and understanding of dynamic maps. To test that, we evaluated whether segmentation agreement was above the chance level and specific to the stimuli. Additionally, we investigated if the ratio of event duration and the duration of its presentation impacts at which points participants gave segmentation responses. To that end, we manipulated the event duration between groups by changing the unit of the displayed timeline but keeping everything else the same. We then tested whether segmentation agreement was higher within each group than between the two groups.

### Methods

#### Participants

We recruited 80 participants (23 women and 57 men) between 19 and 65 years of age (mean age $= 32.15$, $SD = 10.32$) through Prolific (www.prolific.com). All participants reported being fluent in English and having normal or corrected-to-normal vision. No participants had to be excluded for failing the attention checks included in the experiment. All participants received 5.30 £as compensation. Ethics approval for the study series was obtained before we began recruiting participants (LEK 2023/003 of the local ethics committee).

#### Materials

**Fig. 1:**
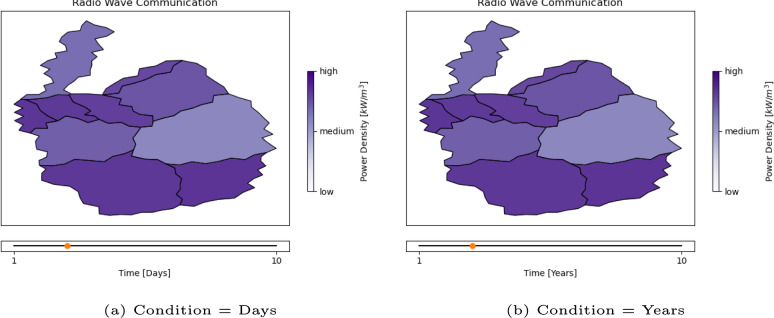
Example stimuli frames for Experiment 1: The animated maps show the development of radio wave communication density over time. Only the unit of the timeline differs between conditions. The title, color scale, and the map itself are kept constant

We prepared 15 videos showing animated maps as stimuli (see Fig. 1b for still images and Example 1A and Example 1B for video examples). Each video lasted 60 s. The first step in creating a map video was to prepare the map outline. For that, a shape was picked from the natural earth data set. To create regions, ten random points were drawn within that shape. Ten regions were formed around the ten random points using the Voronoi algorithm (Aurenhammer and Klein, 2000) from these points. For a more realistic impression, noise was added to the resulting borders.

The second step in creating a map was to determine the data that it should depict. We aimed to create semi-random data dynamics. To that end, we sampled random change rates (slopes) from the interval [-1:1]. Starting from 0, the global value of the map for each second is then determined by applying the slope. Further, we randomly drew the number of times this slope was to change throughout the animation [minimum 5, maximum 10 times]. At which second the changes of slope were applied was also determined randomly. These global values were used to create a timeline of values for each of the regions. First, global values were scaled to the range [0,1]. After that, noise drawn from a normal distribution with a mean of 0 and a standard deviation of 0.1 was added for each region to create ten distinct time series from the global one. Finally, the values were again scaled to range between 0 and 1. The resulting values were depicted on the maps using the saturation-based color scale Purples (Hunter, 2007).

The legend for the color scale was added to the right side of the map. It had the entries “low,” “medium,” and “high,” and the description “Power Density [kW/m^3^].”

A timeline ranging from 1 to 10 was shown at the bottom of the map. Depending on the condition, the title of the timeline was “Time [Years]” or “Time [Days].” The map videos were animated with 24 frames per second.

To ensure optimal viewing conditions, the experiment was presented in a window with a minimum width of 1000 pixels and a minimum height of 600 pixels

#### Design

The between-subjects experiment assigned participants to one of two conditions: In the short time-span condition, the label of the axis of the maps read “Days”; in the long time-span condition, it read “Years.” Otherwise, the stimuli shown in the two conditions did not differ in any way.

To test whether segmentation agreement was above chance level and specific to the stimuli, segmentation agreement was calculated and evaluated separately for the two experimental groups. To calculate it, first of all, button presses occurring less than 500 ms after the previous one are excluded. Then, the button presses are transformed into a time series of 1-second bins containing information on whether a participant gave a segmentation response in a given bin or not (Papenmeier et al., 2019). That time series is correlated with the group norm of all other participants, specifically the percentage of the other participants who gave a segmentation response in each bin. To avoid confounding by the number of button presses, this observed correlation $r_{obs}$, and it was scaled using $r_{min}$ and $r_{max}$, which denote the minimum and maximum possible correlations given the number of button presses. The resulting measure (Eq. 1) ranges from 0 to 1.

1 $$\begin{aligned} \textit{segmentation agreement } = \frac{r_{obs} - r_{min}}{r_{max}- r_{min}} \end{aligned}$$

#### Procedure

The participants opted to partake in the study on the platform Prolific and were then forwarded to the experiment itself. They were presented with the detailed study information and gave their informed consent.

Afterward, they read the instructions for the study. The first part of the introduction introduced the context for the maps that were shown later in the experiment. Specifically, participants were asked to imagine themselves as part of a mission to explore distant solar systems in search of intelligent life. To that end, they had to review data concerning alien communication behavior measured in radio waves for different areas of a foreign planet. Depending on the condition they were assigned to, they were told that the data they were about to review was collected over the span of the last ten days or the last ten years. The segmentation task was introduced.

Before advancing to the main part of the experiment, participants had to answer an instruction comprehension check consisting of two questions (see A), one of which addressed the unit of the timeline to draw the participants’ attention to it. If a person answered incorrectly to either of the instruction comprehension check questions after two attempts, they could not participate in the study.

In the main part of the experiment, participants saw the 15 map stimuli in a randomized order and fulfilled the segmentation task. Each map was displayed once. Between maps, a pause screen was presented. Participants could pace the experiment themselves by deciding when to advance to the next map.

Three attention check trials were presented to the participants in between the experimental trials. They were included to ensure that participants were actually observing the screen throughout the experiment. An attention check trial started just as a regular trial, but after 10 s, the map itself was replaced with the text “This is an attention check. Please press the letter key k.” If participants did not press the letter key k within 10 s, the attention check was considered failed.

After completing all 15 maps, participants were given two open questions: “Please describe which properties of the maps influenced your decisions when to press the spacebar key most strongly.” and “During the review of the maps, did you think about what may be causing the changes in radio wave density over time? If yes, please describe what you thought was driving the change.”

At the end of the experiment, participants were sent back to the homepage of Prolific for compensation.

### Results

#### Descriptive

On average, participants gave a segmentation response 11.01 times ($SD = 9.66$) per stimulus. We observed a mean of 12.21 ($SD = 9.67$) responses per stimulus in the “Days” and 9.82 ($SD = 9.51$) in the “Years” condition. The mean duration of the identified event units across all stimuli was 5.10 seconds ($SD = 6.11$). Looking at the two conditions separately, we find a mean event unit duration of 4.64 ($SD = 5.52$) seconds in the “Days” and 5.67 seconds ($SD = 6.72$) in the “Years” condition. Segmentation agreement was calculated separately for the two experimental groups. In the “Days” condition, the mean segmentation agreement was 0.64 ($SD = 0.12$), and in the “Years” condition, it was 0.61 ($SD = 0.13$).

#### Segmentation agreement is above chance level

**Fig. 2:**
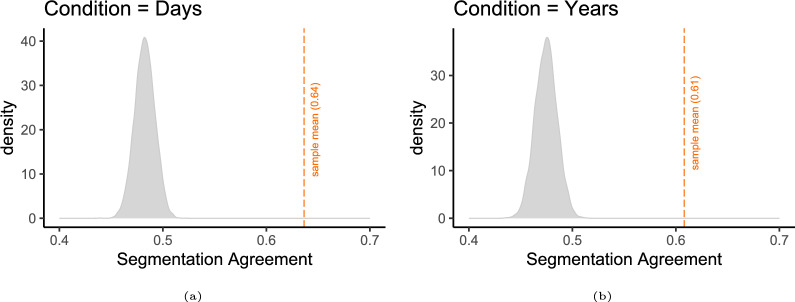
Experiment 1: Density distributions of bootstrapped null segmentation agreement estimates under the assumption of randomly timed key presses for the days (left) and years (right) experimental conditions. The x-axis represents segmentation agreement values, while the y-axis indicates density across 10,000 bootstrap iterations. Vertical dashed lines represent the mean observed segmentation agreement in each condition (days: 0.64, years: 0.61). The probability of observing the sample mean under a random segmentation process was computed using a bootstrap procedure, revealing that observed segmentation agreement was significantly higher than chance level ($p <.001$)

To evaluate whether the segmentation agreement found among participants exceeded the chance level, we bootstrapped a null distribution *Pr*(*x*) of the mean sample segmentation agreement, assuming randomly timed key presses (Fig. 2a). For 10, 000 iterations, the binned time series were randomly permutated before computing the segmentation agreement. Consequently, the number of responses remained identical to the observed data, while the temporal placement was randomized. We can calculate the probability of observing the mean segmentation agreement of our sample if the underlying segmentation process was indeed a random process.

For the experiment group “Days,” we find a mean random sample agreement of 0.48 ($SD = 0.0097$) across all bootstrap iterations. The 2.5% quantile is 0.463, and the 97.5% quantile is 0.502. The difference between the mean random sample agreement and the mean observed agreement is 0.16.

The probability of a difference being as large or larger as the one we observe is calculated as $p = P(X < (0.48 - 0.16)) + (1 - P(X> (0.48 + 0.16)))$.

With $p <.001$, we can conclude that the segmentation agreement we observed in our sample is significantly higher than chance level.

In the years-condition, we find a mean random sample agreement of 0.47 ($SD = 0.01$), resulting in a difference to the sample mean of 0.134 ($p < .001$). Hence, segmentation agreement can be concluded to be above chance level for the second experimental group (years) as well (Fig. 2b).

#### Segmentation is specific to the stimulus

To test whether the segmentation agreement is not just above the chance level but also specific to each stimulus, we compared the observed segmentation agreement (same stimulus) to the segmentation agreement across stimuli. This comparison allowed us to determine whether above-chance agreement reflected reliable, stimulus-tied segmentation rather than general non-random response patterns (e.g., avoiding the first seconds of a stimulus or not clustering all segmentation responses in a short time window). For the cross-stimuli baseline, we took each participant’s segmentation time series for a given stimulus and correlated it with the group norm for each of the other 14 stimuli, yielding 14 across-stimuli agreement values per participant–stimulus pair. These calculations were performed separately for the two experimental groups.

**Fig. 3:**
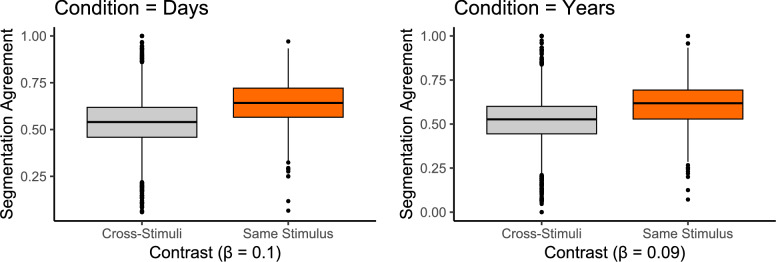
Experiment 1: Boxplots show segmentation agreement scores for cross-stimuli (gray) and same-stimulus (orange) contrasts. Boxes indicate interquartile range (IQR), with black dots representing outliers (outside 1.5xIQR above Q3 or below Q1). Higher segmentation agreement is observed for same stimulus in both conditions. $\beta$-values represent unstandardized parameter estimates from mixed-effects models fit to each condition separately

Linear mixed-effects models were fit to the resulting data, with segmentation agreement as the dependent variable and the type of stimuli contrast (same stimulus vs. cross-stimuli) as the fixed effect, including random intercepts for participants and stimuli. The fixed effect, therefore, tests the difference between segmentation agreement for the same stimulus (standard segmentation agreement) and segmentation agreement with responses given to the other stimuli. The results are reported in Tables 1 and 2. We find that segmentation agreement is significantly higher for the same-stimulus than for the across-stimuli contrast both in the days- ($\beta = 0.099, 95\% \text { CI } [0.089, 0.108], t(8781) = 19.70, p < .001, d = 0.790$) and in the years-condition ($\beta = 0.0864, 95\% \text { CI } [0.076, 0.096], t(8796) = 16.80, p < .001, d = 0.675$), indicating that the segmentation process we measured was specifically elicited by the different stimuli we presented (Fig. 3).

**Table 1:** Results of the linear mixed-effects model for segmentation agreement in Experiment 1. The fixed effect was the contrast: same- versus across-stimuli agreement (condition = days)

Fixed effects							
	$\beta$	SE	95% CI		t	df	p
			Lower	Upper			
Intercept	0.537	0.008	0.521	0.553	65.94	41.99	$<.001$
Contrast	0.099	0.005	0.089	0.108	19.70	8781	$<.001$

Random effects							
	Var						SD
Participant (Intercept)	0.0013	0.0366					
Stimulus (Intercept)	0.0005	0.0216					
Residual	0.0138	0.1172					

Model fit							
	Marginal	Conditional					
$R^2$	0.0374	0.149					

p values and dfs for fixed effects were calculated using Satterthwaite’s approximations. Confidence intervals were calculated using the Wald method. Model equation: segmentation agreement $\sim$ contrast + (1 | participant) + (1 | stimulus)

**Table 2:** Results of the linear mixed-effects model for segmentation agreement in Experiment 1. The fixed effect was the contrast: same- versus across-stimuli agreement (condition = years)

Fixed effects							
	$\beta$	SE	95% CI		t	df	p
			Lower	Upper			
Intercept	0.521	0.008	0.505	0.537	64.69	43.82	$<.001$
Contrast	0.0864	0.005	0.076	0.096	16.80	8796	$<.001$

Random effects							
	Var	SD					
Participant (Intercept)	0.0014	0.0371					
Stimulus (Intercept)	0.0004	0.0207					
Residual	0.0146	0.1207					

Model fit							
	Marginal	Conditional					
$R^2$	0.0276	0.1349					

p values and dfs for fixed effects were calculated using Satterthwaite’s approximations. Confidence intervals were calculated using the Wald method. Model equation: Segmentation Agreement $\sim$ contrast + (1 | Participant) + (1 | Stimulus)

#### No effect of timeline unit

**Fig. 4:**
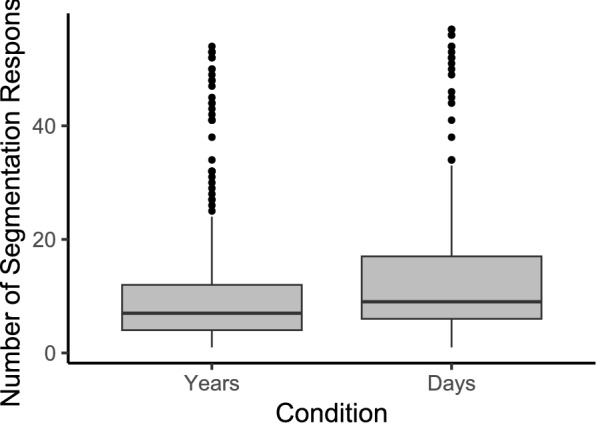
Experiment 1: Boxplots show number of segmentation responses by condition. Boxes indicate interquartile range (IQR), with black dots representing outliers (outside 1.5xIQR above Q3 or below Q1). The median number of responses was 7 in the years-condition and 9 in the days-condition, but the difference was not statistically significant

We tested whether the unit of the maps’ timeline impacted segmentation grain by modeling the number of segmentation responses given as a linear mixed-effects model with timeline unit as the fixed effect and including random intercepts for participants and stimuli. The results are reported in Table 3. We find no effect of the timeline unit on the number of segmentation responses given by participants ($\beta = 2.338, 95\% \text { CI } [-1.63, 6.30], t(78.0) = 1.156, p = .251$), as shown in Fig. 4.

**Table 3:** Results of the linear mixed-effects model for number of segmentation responses in Experiment 1. The fixed effect was the timeline unit condition (days vs years, between-subjects)

Fixed effects							
	$\beta$	SE	95% CI		t	df	p
			Lower	Upper			
Intercept	9.700	1.457	6.85	12.55	6.659	83.05	$<.001$
Time Unit	2.338	2.023	$-$1.63	6.30	1.156	78.00	.251

Random effects							
	Var	SD					
Participant (Intercept)	81.047	9.003					
Stimulus (Intercept)	1.131	1.064					
Residual	11.666	3.416					

Model fit							
	Marginal	Conditional					
$R^2$	0.014	0.877					

p values and dfs for fixed effects were calculated using Satterthwaite’s approximations. Confidence intervals were calculated using the Wald method. Model equation: number of segmentation responses $\sim$ time unit + (1 | participant) + (1 | stimulus)

**Fig. 5:**
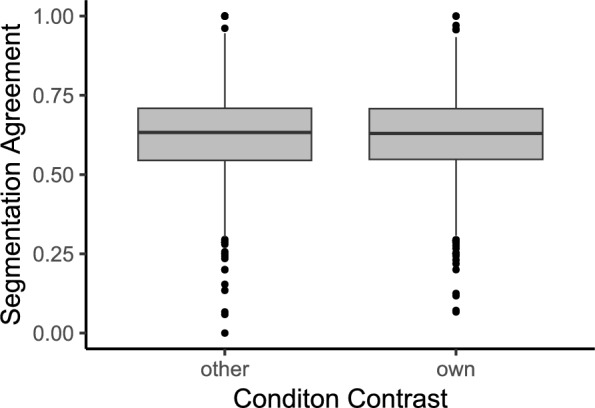
Experiment 1: Boxplots show segmentation agreement computed for each participant with the participants in their own vs the other experimental group. This corresponds to participants having seen the same timeline unit [days or years] or the other. Boxes indicate interquartile range (IQR), with black dots representing outliers (outside 1.5xIQR above Q3 or below Q1). We observe no difference between the two types of contrasts, indicating that the timeline unit did not impact segmentation behavior

Additionally, we tested whether boundary placement was influenced by the timeline unit. For each participant and stimulus, we computed the observed segmentation agreement with their own experimental group (as in the other analyses) and the segmentation agreement with the other experimental group (see Fig. 5). A linear mixed-effects model was fit to the resulting data, using type of contrast (same stimulus vs across stimuli) as the fixed effect and including random intercepts for participants and stimuli. The results are reported in Table 4. The model does not indicate that people agree more with people viewing the same timeline unit than with people viewing a different timeline unit ($\beta = -5.91\times 10^{-4}, 95\% \text { CI } [-0.009, 0.008], t(2261) = 1.129, p = .895$)

**Table 4:** Results of the linear mixed-effects model for segmentation agreement by group of Experiment 1: The fixed effect group describes whether segmentation agreement was calculated for the respective participant’s own or the other experimental group. One group was assigned to the years- and the other to the days-condition, altering the unit of the timeline of the maps they observed

Fixed effects							
	$\beta$	SE	95% CI		t	df	p
			Lower	Upper			
Intercept	0.621	0.012	0.597	0.645	50.67	33.94	$<.001$
Group [Own]	-5.91e$-$4	4.48e$-$3	$-$0.009	0.008	-0.132	2261	$0.895$

Random effects							
	Var	SD					
Participant (Intercept)	0.004	0.064					
Stimulus (Intercept)	0.001	0.036					
Residual	0.012	0.109					

Model fit							
	Marginal	Conditional					
$R^2$	5.06e$-$6	0.316					

p values and dfs for fixed effects were calculated using Satterthwaite’s approximations. Confidence intervals were calculated using the Wald method. Model equation: segmentation agreement $\sim$ comparison group + (1 | participant) + (1 | stimulus)

#### Exploratory: a positive trend increases segmentation agreement

While reviewing which stimuli were fit with high or low random intercepts by the models, we noticed that maps depicting increasing values elicited higher segmentation agreement. (Example 1A shows a strong increase over time and elicited the highest average segmentation agreement of 0.67. Example 1B, showing a strong decrease in values over time, showed the lowest average segmentation agreement at 0.57.) An increasing or positive trend meant that all regions began at lower saturation levels and ended at higher saturation levels overall, whereas a decreasing or negative trend was the reverse. Although trends were defined by the difference between the average value in the last and first frame, they were not necessarily monotonic—an “increasing” map could contain short phases of decrease, and a “decreasing” map could contain short phases of increase. In this experiment, the trend was not varied systematically. The start values were selected randomly, as were the trends and their duration. To explore the apparent relationship between overall map characteristics and segmentation agreement, we fit an exploratory model including three fixed effects characterizing the values depicted by the maps: First, the trend of the map was defined as the average value depicted in the last frame of the animation minus the average value of the first frame. Second, the sum of values is defined as the sum of the average map values across all frames. Third, the maximal slope was the largest absolute change in average value between two consecutive frames. These were not systematically varied but randomly generated during stimuli production.

**Table 5:** Results of the exploratory linear mixed-effects model for segmentation agreement in Experiment 1: fixed effects include trend, sum of values, and maximal slope, along with their two-way and three-way interactions

Fixed effects							
	$\beta$	SE	95% CI		t	df	p
			Lower	Upper			
Intercept	0.591	0.038	0.510	0.672	15.48	12.03	.001
Trend	0.037	0.005	0.026	0.049	6.94	11.07	$<.001$
Sum values	0.002	0.002	$-$0.003	0.006	0.87	11.20	.403
Max slope	0.007	0.079	$-$0.154	0.168	0.09	11.07	.928

Random effects							
	Var	SD					
Participant (Intercept)	0.0043	0.065					
Stimulus (Intercept)	0.0001	0.011					
Residual	0.0117	0.108					

Model fit							
	Marginal	Conditional					
$R^2$	0.050	0.309					

p values and dfs for fixed effects were calculated using Satterthwaite’s approximations. Confidence intervals were calculated using the Wald method. Model equation: segmentation agreement $\sim$ trend + summed_values + maximal_slope + (1 | subject_id) + (1 | stimulus_id)

**Fig. 6:**
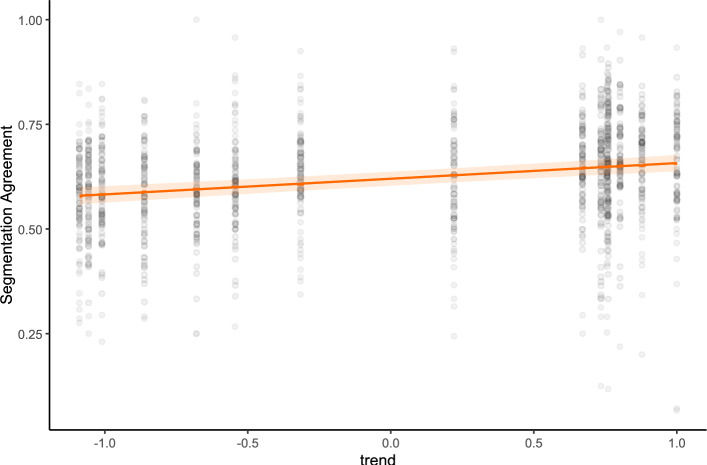
Experiment 1: The line represents predicted mean segmentation agreement as a function of the trend depicted on the map derived from a mixed-effect model. Shaded areas indicate the 95 % confidence interval. Points represent observed data points

The results noted in Table 5 indicate that, indeed, the overall trend of the map predicts higher segmentation agreement, as shown in Fig. 6.

### Discussion

These findings indicate that participants’ segmentation responses were consistently above chance level in both experimental groups, suggesting a robust ability to identify meaningful event boundaries within dynamic maps. They further show that the segmentation patterns are unique for each map and not primarily driven by approaches to segment based on the accompanying timeline or other shared features of the stimuli. This specificity underscores the relevance of stimulus characteristics in shaping segmentation processes. Moreover, the mean agreement scores between participants of 0.64 and 0.61 fit well to the levels of agreement reported for other stimuli classes: Across 1000 bootstrap iterations of segmentation data on commercials and everyday scenes, Sasmita and Swallow (2022) found mean agreement values between 0.47 for coarse segmentation of commercials and 0.69 for fine segmentation for everyday scenes. The results support the idea that segmentation is a relevant process in perceiving and understanding dynamic thematic maps, which warrants further investigation. No measurable effects of the timeline manipulation on segmentation behavior were found. This could indicate that map viewers assume that maps use an appropriate time scale for the depicted events and adjust their segmentation accordingly. Another possibility is that they simply disregard the relationship between represented time and viewing time, basing their segmentation on other available information. Due to the between-subjects manipulation of the timeline unit, it is also plausible that participants paid little to no attention to the timeline, as it was an unchanging and, therefore, less interesting aspect of the maps. The null result, therefore, does not imply that conceptual factors are absent, but that this particular manipulation may not have influenced segmentation under the tested conditions. Going forward, all manipulations of this experimental series use a within-subject design. In exploratory analyses, we found higher segmentation agreement for maps showing an increase in radio wave communication density over time. On the one hand, this pattern could emerge from an interpretation of increases in activity as more meaningful than decreases. On the other hand, increases also mean that the overall saturation of the map increases, which could be perceptually more stimulating than decreasing saturation. As discussed, both conceptual and perceptual properties of stimuli have been shown to drive segmentation behavior (Zacks, 2004; Hard et al., 2006; Kurby and Zacks, 2008). Due to the confounding of visual change and interpretation in the stimulus material, this influence cannot be further differentiated from the data of this experiment but is explored in-depth in the experiments reported next.

## Experiment 2: Framing and color scale

This experiment tests the hypothesis that the positive relationship between a map’s depicted trend and the degree of agreement participants achieve in the segmentation of that map is malleable both by bottom-up effects of the perceptual salience of the trend and by top-down influences of semantic interpretation thereof. To be able to independently manipulate the conceptual interpretation of the depicted change and the perceptual salience of the direction of that change, we introduce a new context for the maps used as stimuli: The maps in this experiment show how different (fictional) insect species on different (also fictional) islands develop regarding their population sizes. The trend was varied systematically, ranging from strongly decreasing to strongly increasing.

The conceptual interpretation of the development of population size was manipulated by framing the species as either invasive or endangered. Through these two different framings, participants were prompted to form opposing expectations and appraisals of the direction in which the population size develops. The perceptual salience of the direction of change was manipulated through the type of color scale used to translate population size to RGB values. As the high-salience condition, saturation-based color scales were used. The direct mapping between population size and saturation makes it easy to perceive the direction of development, and such scales are recommended for unclassed metric data (Battersby and Goldsberry, 2010). In contrast, hue-based scales are used for low-salience condition. They require more effort to mentally map population sizes to different hues and, therefore, make it harder to perceive the direction of the trend.

The operationalized hypothesis is a 3-way interaction between trend, framing, and color scale. Specifically, we expect higher agreement for positive trends in the invasive framing condition and for negative trends in the endangered framing. These combinations match the usual directions of change tied to the categories’ definitions themselves: Invasive species are generally seen as spreading and multiplying, and endangered species as declining. We only expect this pattern of improved agreement for expectation-congruent developments in combination with saturation-based color scales and expect no effects for hue-based color scales.

### Methods

#### Participants

We recruited 125 participants (43 women and 82 men) between 18 and 63 years of age (mean age = 30.56, SD = 9.9) via Prolific. All participants reported being fluent in English and having normal or corrected-to-normal vision. Two participants had to be excluded for failing the attention checks included in the experiment, new participants were recruited to fill the places. All participants received £5.13 as compensation. The sample size of 125 was predetermined by power simulation. It yields a power of 0.8 to detect an effect of the hypothesized three-way interaction of 0.02 at the 5% significance level. Ethics approval for the study series was obtained before we began recruiting participants (LEK 2023/003 of the local ethics committee)

#### Materials

**Fig. 7:**
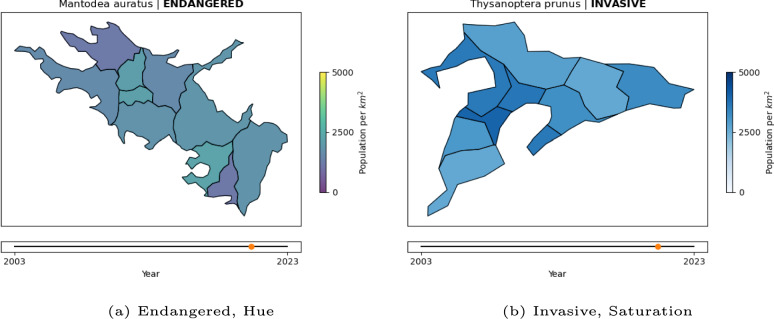
Example stimuli frames for Experiment 2: The animated maps show the development of insect population densities over time. The trend of that development, the framing of the species, and the color scale were varied between maps. Two example combinations are depicted here

In this experiment, we produced 32 maps that each lasted 30 seconds. Figure 7 shows still images illustrating key components. For example videos, see Example 2A showing a trend of 0.43 in a hue scale and Example 2B showing a trend of $-0.43$ in a saturation scale. The map outlines were prepared in the same manner as for the first experiment. The data dynamic to be shown on the map was explicitly manipulated as an independent variable in this experiment rather than being randomized as well as possible, as was the case in the first experiment. The slope of the map—or the trend—was varied in 8 steps from -1 to 1: -1.00 -0.71 -0.43 -0.14 0.14 0.43 0.71 1.00. In this experiment, the trend defines the change of overall values from the map video’s first to the last frame. The start value of the map was drawn randomly from the range of values that still allowed the map values to remain in the range between 0 and 1 after applying the slope. Hence, a map with a slope of -1 can only start with 1, whereas a map with a slope of -0.43 can start at any value between 0.43 and 1.

To this global trend, noise drawn from a normal distribution with a mean of zero and a standard deviation of 0.1 was added to each region. To retain the manipulation of trend, the values of these maps were not scaled to the range $0 - 1$ as were the ones for the first experiment. Instead, values exceeding these limits due to the addition of noise were truncated.

The color scales used to depict the resulting values were explicitly manipulated as an independent variable for this experiment. Specifically, we varied between hue-based and saturation-based color scales. Purples, greens, oranges, and blues were used as the saturation scales. Summer, Winter, Viridis, and Wistia were used as the hue-based scales (Hunter, 2007).

The legend for the color scale was added to the right side of the map. It had the entries “0,” “2500,” and “5000” and the description “Population per km2.” A timeline from 2003 to 2023 was shown at the bottom of the map.

For this experiment, titles were added to the maps to manipulate the third independent variable. Each title contained the name of the made-up insect species whose population density was to be depicted. The species names were produced by ChatGPT (OpenAI, 2024). Next to the species name, the information on whether the insect was considered invasive or endangered was presented to participants—the two values of the independent variable framing.

The map videos were animated with 24 frames per second. Two sets of stimuli, using the same shapes but a different shape assignment to the combination of values of the independent variables, were produced, and each was shown to half of the participants.

The experiment was shown in a window with a minimal width of 1000 pixels and a minimal height of 600 pixels.

#### Design

The experiment used a within-subjects design. The three independent variables were trend, framing, and color scales. Variable expressions (8 trend values, two framings, and two color scales) were combined orthogonally to produce the 32 stimuli.

#### Procedure

The participants came to the online experiment through Prolific, were presented with the relevant information concerning data protection, and consented to participate. Then, they were presented with the study‘s instructions. They were asked to imagine themselves as an insect researcher collecting data on insect population numbers on different remote islands. The role of insects in functioning ecosystems was explicitly stressed. It was explained that the data had been processed to be reviewed in the form of color-coded maps. It was further described that the maps showed the population development of different endangered and invasive species. In order to ensure the framing had similar effects regardless of the prior knowledge of the participants, they were provided with the following definitions of endangered and invasive species:

*Endangered species are plants, animals, or – most interesting to you – insects that are at risk of disappearing forever. This happens because they do not find good places to build nests, there is too much pollution or not enough food, or because other species that shouldn’t be there are causing problems for them. When a species becomes endangered, it means there aren’t many left, and they need help to survive. People and organizations work together to protect them, make sure they have a safe place to live and prevent harm from coming to them.*

*Invasive species are living beings that are not native to a certain place and end up causing trouble. They can be plants, animals, or insects. What makes them invasive is that they don’t have any natural enemies or predators in their new environment. This allows them to grow and reproduce very quickly, which can be harmful to the other plants, animals, and insects that already live there. They take up resources like food and space, and they can make the others sick. It’s important to control invasive s species because they can disrupt the natural balance of the environment.*

The segmentation task itself was explained in the same way as in the previous experiment. Participants were required to pass an instruction comprehension check before beginning the main part of the experiment, consisting of four multiple-choice questions listed in B. The questions tested the participant’s understanding of the task and of the definitions of invasive and endangered species to ensure a successful framing manipulation. Participants had two opportunities to answer correctly; otherwise, they could not participate.

The main block of the experiment consisted of presenting the 32 map stimuli in a randomized order. The participants decided when to progress to the next map. Before each map, an announcement with the following format was shown to draw attention to the framing variation: “The next map shows the population development of *Species* on the *Island*. *Species* is classified as *Framing*.”

In between the experimental trials, seven attention checks were included. Three of those had the same format as the attention checks used previously—a map that changes into the request to press the letter key k. Two attention checks displayed two colors on the screen. Participants had to indicate which corresponded to a higher or lower value in the last map they had seen. The remaining two attention checks asked whether the last map displayed the population development of an endangered or an invasive species.

At the end of the experiment, as in the first experiment, participants were asked which properties of the maps influenced their segmentation decisions and what they thought might be causing the depicted changes. Participants were sent back to the homepage of Prolific, over which they received their compensation.

### Results

Eight participants completed the experiment without giving any segmentation responses to any of the stimuli but passed the attention checks and responded to the final open questions. One person reported not having perceived changes drastic enough to prompt them to press the key and two reported misunderstanding the task. The remaining five participants described the factors leading them to press the key in the open question, leading to the assumption they might have pressed a wrong key, which was not logged, or experienced other technical difficulties. Therefore, data from 117 subjects entered the analysis. A programming error in the experiment led to one stimulus being omitted and another being shown twice instead. The second time the doubled stimulus was shown was removed from the data prior to analyses. The stimulus showing a trend of 1 on a hue scale with invasive framing was unfortunately not seen by participants and, therefore, could not enter the analysis. Of the overall number of 3627 trials ($117 * 31$), 267 were trials in which no response was given and 11 trials contained responses after the video stimulus ended and were excluded due to suspected logging errors. 3349 trials could be used to calculate and analyze segmentation agreement.

On average, participants gave 3.51 segmentation responses ($SD = 2.61$) per stimulus. (Responses occurring within 500 ms of the previous were excluded.) The mean duration of event units was 6.87 seconds ($SD = 6.22$). We observed a mean segmentation agreement of 0.59 ($SD = 0.2$).

**Fig. 8:**
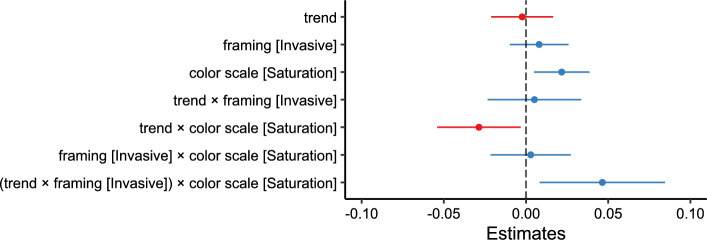
Experiment 2: Estimated effects of trend, framing (invasive), color scale (saturation), and their interactions on segmentation agreement, as derived from a mixed-effects model. The x-axis represents unstandardized parameter estimates ($\beta$), with positive values indicating increased segmentation agreement and negative values indicating decreased agreement. Error bars represent 95% confidence intervals. Estimates with confidence intervals that include zero suggest non-significant effects. Full model details, including fixed and random effects specifications, are reported in Table 6

We fit a linear mixed-effects model with segmentation agreement as the outcome variable, including trend, framing, and color scale, and their two- and three-way interactions as fixed effects, random intercepts, and random slopes for trend, framing, and color scale per participant. The model is reported in Table 6 and Fig. 8.

**Table 6:** Results of the linear mixed-effects model for segmentation agreement in Experiment 2. Fixed effects include trend [-1:1], framing [endangered vs. invasive], and color scale [hue vs. saturation], along with their two-way and three-way interactions

Fixed effects							
	$\beta$	SE	95% CI		t	df	p
			Lower	Upper			
Intercept	0.569	0.010	0.549	0.590	54.72	130.3	$<.001$
Trend	$-$0.002	0.010	$-$0.021	0.017	$-$0.243	834.6	.808
Framing (Invasive)	0.008	0.009	$-$0.010	0.026	0.88	2918.0	.381
Color Scale (Saturation)	0.022	0.009	0.005	0.039	2.52	387.6	.012
Trend:Framing	0.005	0.015	$-$0.023	0.034	0.35	3005.0	.725
Trend:Color Scale	$-$0.029	0.013	$-$0.054	$-$0.003	$-$2.21	3006.0	.027
Framing:Color Scale	0.003	0.012	$-$0.022	0.027	0.23	3007.0	.819
Trend:Framing:Color Scale	0.046	0.019	0.008	0.085	2.39	3008.0	.017

Random effects							
	Var	SD	Correlation				
Participant (Intercept)	0.00829	0.0911					
Part. (Trend)	9.63e$-$4	0.0310	0.61				
Part. (Framing)	7.81e$-$6	0.0028	$-$0.32	$-$0.74			
Part. (Color Scale)	2.01e$-$4	0.0142	$-$0.20	$-$0.69	0.99		
Residual	0.0316	0.1777					

Model fit							
	Marginal	Conditional					
$R^2$	0.008	0.216					

*Note.* p values and dfs for fixed effects were calculated using Satterthwaite’s approximations. Confidence intervals were calculated using the Wald method. Model equation: segmentation agreement $\sim$ trend + framing + color_scale + trend:framing + trend:color_scale + framing:color_scale + trend:framing:color_scale + (trend + framing + color_scale | participant)

**Table 7:** Marginal effects of trend on segmentation agreement depending on framing and color scale in Experiment 2

Marginal effects of trend							
Framing	Color	AME	SE	95% CI		*z*	*p*
				Lower	Upper		
Endangered	Hue	$-$0.001	0.010	$-$0.020	0.017	$-$0.147	.883
Endangered	Saturation	$-$0.030	0.010	$-$0.049	$-$0.011	$-$3.134	.002
Invasive	Hue	0.004	0.012	$-$0.018	0.027	0.338	.736
Invasive	Saturation	0.021	0.010	0.003	0.040	2.229	.026

In comparison with hue scales, saturation scales elicited higher segmentation agreement ($\beta = 0.022, 95\% \text { CI } [0.005, 0.039], t(387.6) = 2.52, p = .012, d = 0.107$).

**Fig. 9:**
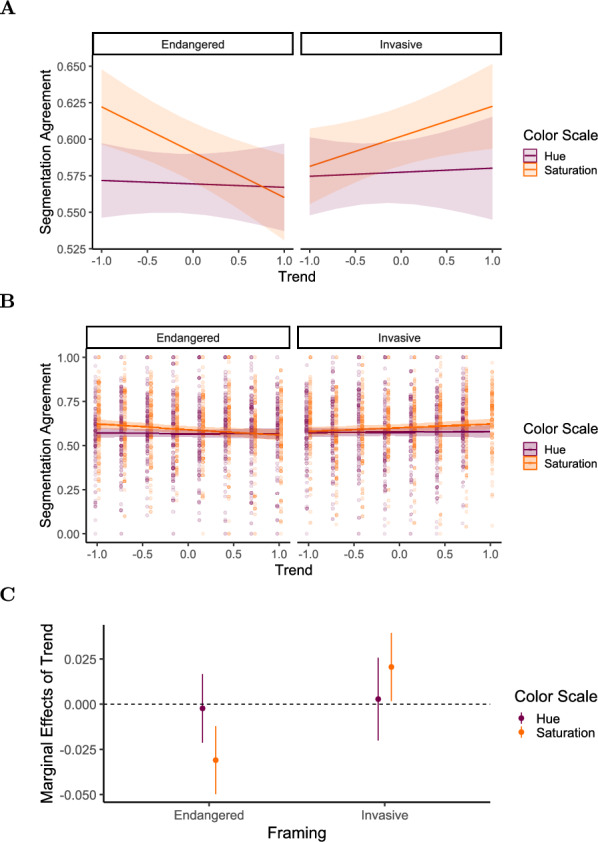
Experiment 2: Predicted segmentation agreement values as a function of trend, framing (endangered vs. invasive), and color scale (hue-based [purple/dark gray] vs. saturation-based [orange/light gray]), derived from a mixed-effects model. (A) Model-predicted mean segmentation agreement across levels of trend, framing, and color scale. Shaded areas represent 95% confidence intervals. (B) The same predicted values overlaid on observed data points, (C) Marginal effects of trend on segmentation agreement across framing and color scale conditions. Points represent model-estimated effects, with error bars indicating 95% confidence intervals. Full model specifications and significance tests are detailed in Tables 6 and 7

We further find a significant interaction effect between trend, framing, and color scale ($\beta = 0.046, 95\% \text { CI } [0.008, 0.084], t(3008.0) = 2.39, p = .017, d = 0.147$) on segmentation agreement. Specifically, the marginal effect of trend (Table 7) on segmentation agreement is negative for the endangered condition $(AME = -0.030, 95\% \text { CI }[-0.049,-0.011], p =.002)$ and positive for the invasive condition $(AME = 0.021, 95\% \text { CI } [0.003,0.04], p =.026)$, but only in combination with the saturation scale. When a hue scale is used, the marginal effect of trend does not differ between the two framing conditions (see Fig. 9).

In a post hoc analysis, we fit an additional linear mixed-effects model with the number of segmentation responses as the outcome variable, using the same fixed and random effect structure as for the segmentation agreement model.

**Fig. 10:**
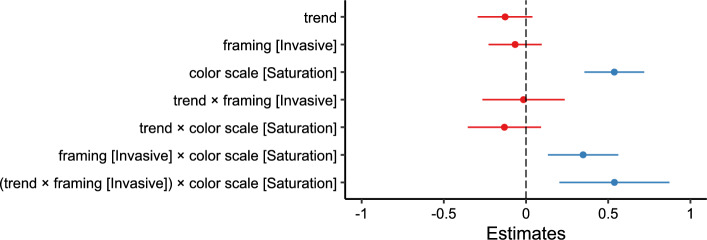
Experiment 2: Estimated effects of trend, framing (invasive), color scale (saturation), and their interactions on the number of segmentation responses, as derived from a mixed-effects model. The x-axis represents unstandardized parameter estimates ($\beta$), with positive values indicating higher segmentation frequency and negative values indicating decreased frequency. Error bars represent 95% confidence intervals. Estimates with confidence intervals that include zero suggest non-significant effects. Full model details, including fixed and random effects specifications, are reported in Table 8

The model is reported in Table 8 and Fig. 10.

**Fig. 11:**
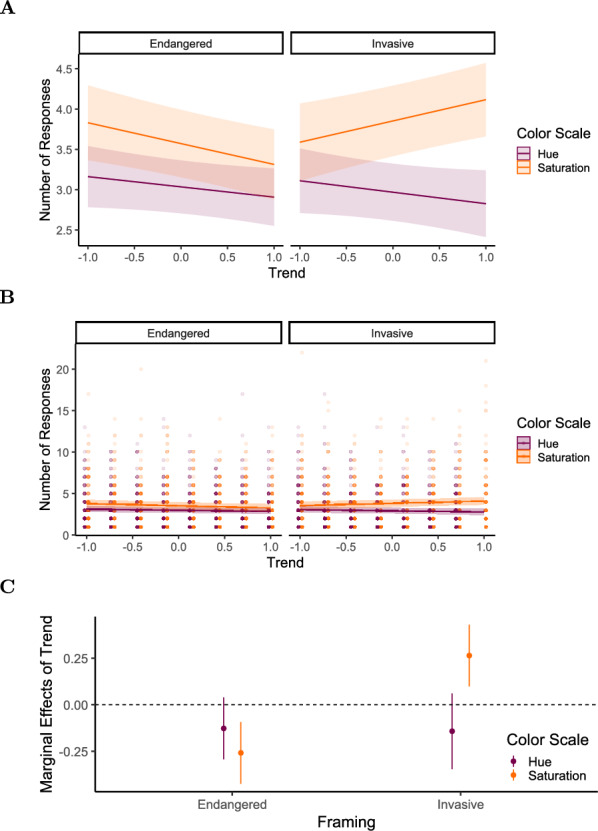
Experiment 2: Predicted number of segmentation responses as a function of trend, framing (endangered vs. invasive), and color scale (hue-based [purple/dark gray] vs. saturation [orange/light gray]), derived from a mixed-effects model. (A) Model-predicted mean number of segmentation responses across levels of trend, framing, and color scale. Shaded areas represent 95% confidence intervals. (B) The same predicted values overlaid on observed data points, (C) Marginal effects of trend on number of segmentation responses across framing and color scales conditions. Points represent model-estimated effects, with error bars indicating 95% confidence intervals. Full model specifications and significance tests are detailed in Tables 8 and 9

Saturation scales elicited more segmentation responses than hue scales ($\beta = 0.537, 95\% \text { CI } [0.355, 0.719], t(242.8) = 5.794, p = <.001, d = 0.222$). Furthermore, we find a significant interaction effect of trend, framing, and color scale ($\beta = 0.538, 95\% \text { CI } [0.203, 0.872], t(3031.1) = 3.151, p = .002, d = 0.222$). Mirroring the result pattern for segmentation agreement, the marginal effect of trend (Table 9) on number of segmentation responses is negative for the endangered condition $(AME = -0.263, 95\% \text { CI } [-0.429,-0.097], p =.002)$ and positive for the invasive condition $(AME = 0.260, 95\% \text { CI } [0.094,0.425], p =.026)$, when combined with the saturation scale. For the hue scale, the marginal effects of the trend are not estimated to differ between the two framing conditions (see Fig. 11).

**Table 8:** Results of the linear mixed-effects model for number of segmentation responses in Experiment 2. Fixed effects include trend [-1:1], framing [endangered vs. invasive], and color scale [hue vs. saturation], along with their two-way and three-way interactions

Fixed effects							
	$\beta$	SE	95% CI		t	df	p
			Lower	Upper			
Intercept	3.034	0.167	2.706	3.362	18.129	122.096	<.001
Trend	$-$0.127	0.085	$-$0.294	0.039	$-$1.498	906.102	.135
Framing (Invasive)	$-$0.066	0.082	$-$0.228	0.095	$-$0.805	1038.607	.421
Color Scale (Saturation)	0.537	0.093	0.355	0.719	5.794	242.823	<.001
Trend:Framing	$-$0.015	0.128	$-$0.265	0.235	$-$0.119	3031.659	.905
Trend:Color Scale	$-$0.131	0.114	$-$0.354	0.091	$-$1.158	3025.561	.247
Framing:Color Scale	0.347	0.109	0.133	0.562	3.179	3035.009	.001
Trend:Framing:Color Scale	0.538	0.171	0.203	0.872	3.151	3031.118	.002

Random effects							
	Var	SD	Correlation				
Participant (Intercept)	2.93776	1.7140					
Participant (Trend)	0.08513	0.2918	$-$0.27				
Participant (Framing)	0.04431	0.2105	0.54	0.67			
Participant (Color Scale)	0.34789	0.5898	0.84	$-$0.43	0.27		
Residual	2.42842	1.5583					

Model fit							
$R^2$	0.0221	0.6458					

*Note.* p values and dfs for fixed effects were calculated using Satterthwaite’s approximations. Confidence intervals were calculated using the Wald method. Model equation: number of segmentation responses $\sim$ trend + framing + color_scale + trend:framing + trend:color_scale + color_scale:framing + trend:color_scale:framing + (trend + framing + color_scale | subj_id)

**Table 9:** Marginal effects of trend on number of segmentation responses depending on framing and color scale in Experiment 2

Marginal effects of trend							
Framing	Color	AME	SE	95% CI		*z*	*p*
				Lower	Upper		
Endangered	Hue	$-$0.131	0.085	$-$0.298	0.035	$-$1.55	.122
Endangered	Saturation	$-$0.263	0.085	$-$0.429	$-$0.097	$-$3.11	.002
Invasive	Hue	$-$0.151	0.103	$-$0.352	0.051	$-$1.47	.142
Invasive	Saturation	0.260	0.085	0.094	0.426	3.07	.002

### Discussion

In line with the hypothesis, we observe a significant 3-way interaction between trend, framing, and color scale. Participants did agree more strongly in their segmentation when the framing and the depicted trend matched (positive trend—invasive, negative trend—endangered) than when it did not. As expected, this effect of the framing on the relationship between trend and segmentation agreement was only present if the color scale provided high salience for the direction of the trend. Exploratory analyses showed that participants not only agreed more strongly but also segmented more frequently when the observed trend and the framing were congruent and the direction of change salient.

The results demonstrate that the segmentation of dynamic maps is driven both by conceptual and perceptual features. The found pattern that conceptual and perceptual influences can moderate the effect of the other on segmentation generalizes prior findings (Zacks, 2004, 2020) to the special case of dynamic maps.

Additionally, we found higher agreement for saturation than hue-based color scales. This effect supports well-known design principles (Battersby and Goldsberry, 2010) with segmentation agreement as a novel measure to assess not only the individual but also the shared understanding of dynamic maps. Possibly, the fact that the saturation scales provide more pronounced changes in luminance and therefore might elicit more apparent motion (Mather, 1984) contributed to this result.

The improved agreement for saturation in comparison with hue-based color scales also highlights a limitation of this experiment: The low-salience condition violates established design principles. In other words, the hue condition might not just have provided low salience of the direction of change but hindered participants from perceiving it to a degree not representative of (well-made) real-world examples. The next experiment was therefore designed to test if the results are reproducible with an ecologically more valid salience manipulation.

## Experiment 3: Framing and spatial pattern

Every map creator can optimize the salience of change through appropriate choices, e.g., the color scale. Other sources of salience (or lack thereof) are out of their control, namely the spatiotemporal pattern of change themselves. In experiment 2, the maps’ underlying trend was spatially uniform, meaning that all 10 map regions followed the same trend and only the added noise distinguished their development over time. This experiment is designed to take one step further toward the spatiotemporal complexity encountered in data collected in the real world. In short, the aim is to replicate the interaction of trend, framing and salience using a different manipulation of salience: The spatial pattern of change. Spatiotemporal change can be difficult to notice (Cybulski, 2022). To create two levels of change salience, we produced maps in which only a subset of regions were affected by the trend while the remainder kept a stable population size plus noise. In this experiment, all maps used the saturation-based color scales to visualize changes. The changing regions were either clustered together for the high-salience condition or distributed across the map for the low-salience condition.

The operationalized hypothesis is a 3-way interaction between trend, framing, and spatial pattern. Specifically, we expect higher agreement for positive trends in the invasive framing condition and for negative trends in the endangered framing. We expect this pattern to be more pronounced for maps depicting spatially clustered change than for maps depicting spatially distributed change.

### Methods

#### Participants

We recruited 176 participants (56 women, 118 men, 2 non-disclosed) between 18 and 71 years old (mean age = 33.79, $SD = 11.81$) via Prolific. All participants reported being fluent in English and having normal or corrected-to-normal vision. Three participants had to be excluded for failing the attention checks included in the experiment, new participants were recruited to fill the places. All participants received £5.13 as compensation. The sample size of 175 was predetermined by power simulation based on the prior results. It yields a power of 0.85 to detect an effect of the hypothesized three-way interaction of 0.02 at the 5% significance level. Three participants completed the experiment twice for unknown reasons. The data from the respective second runs were excluded from analyses. Ethics approval for the study series was obtained before we began recruiting participants (LEK 2023/003 of the local ethics committee)

#### Materials

**Fig. 12:**
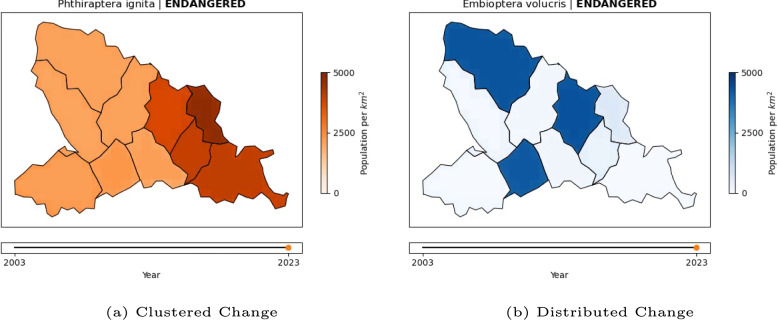
Example stimuli frames for Experiment 3: The animated maps show the development of insect population densities over time. The trend of that development, the framing of the species, and the spatial patterns of change were varied between maps. The examples here show the last frame of two animated maps, demonstrating the spatial pattern manipulation

In this experiment, we again produced 32 maps with a duration of 30 seconds. Figure 12 shows still images of distributed and clustered regions. See Example 3A for a video example of distributed change and Example 3B for clustered change. The slope of the map—or the trend—was varied in 4 steps from -1 to 1: -1.0 - 0.5 0.5 1.0. In this experiment, the trend defines the change of a selected subset of map regions between the first and the last frame—as opposed to the whole map in the previous experiment. 3 or 4 (this variability was included to mask the manipulation of spatial pattern) out of the 10 regions were selected according to the spatial pattern. In both conditions, a random first region was selected. In the “Clustered” condition, the remaining regions were selected randomly but under the constraint that they must share borders with at least one region already selected. In the “Distributed” condition, the remaining regions were selected randomly but under the constraint that they must not share borders with any region already selected—if no regions could fulfill that requirement, a random region was selected instead.

Again, noise drawn from a normal distribution with a mean of zero and a standard deviation of 0.1 was added to each of the 10 regions. In this experiment, the data was depicted using only the saturation scales. The map titles, including the framing manipulation, as well as the legends and frame rates, were identical to the previous experiment.

#### Design

The experiment used a within-subjects design. The three independent variables were trend, framing, and spatial pattern. Variable expressions (four values of trend, two framings, and two spatial patterns) were combined orthogonally, once with 3 and once with 4 changing regions to produce the 32 stimuli. The varying number of changing regions was included to mask the pattern manipulation.

#### Procedure

The procedure was identical to the one used in experiment 2.

### Results

Of the overall number of 5632 trials ($176 * 32$), 395 were trials in which no response was given and 13 trials contained responses given after the video stimulus ended and were excluded due to suspected logging errors. 5224 trials could be used to calculate and analyze segmentation agreement.

**Fig. 13:**
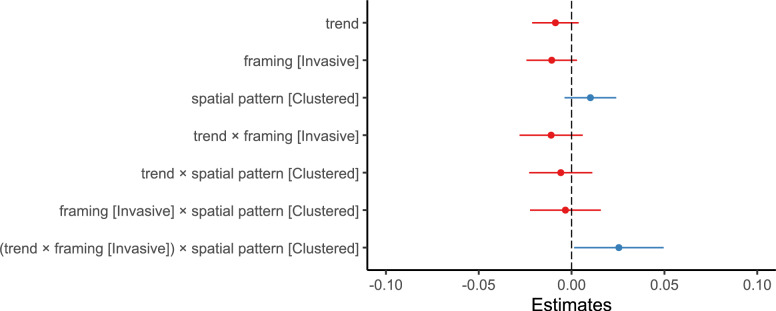
Experiment 3: Estimated effects of trend, framing (invasive), spatial pattern (clustered), and their interactions on segmentation agreement, as derived from a mixed-effects model. The x-axis represents unstandardized parameter estimates ($\beta$), with positive values indicating increased segmentation agreement and negative values indicating decreased agreement. Error bars represent 95% confidence intervals. Estimates with confidence intervals that include zero suggest non-significant effects. Full model details, including fixed and random effects specifications, are reported in Table 10

**Fig. 14:**
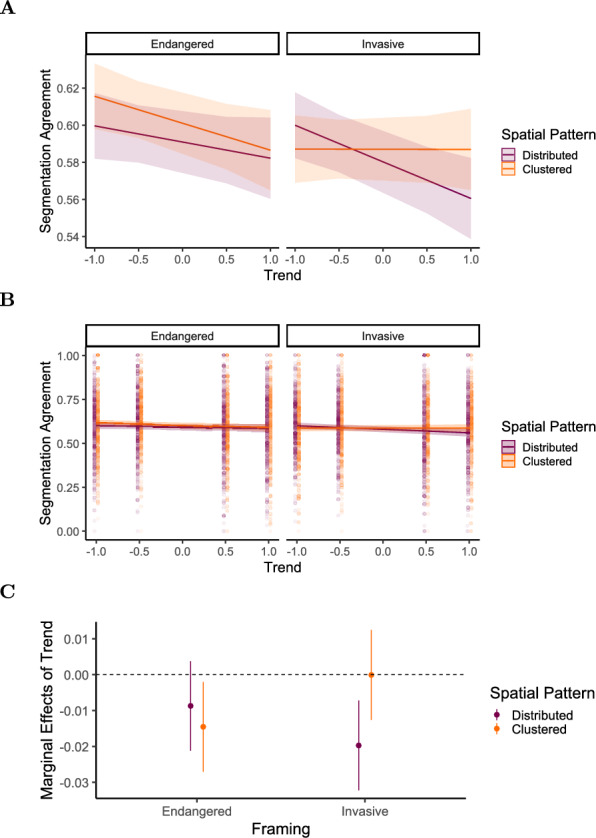
Experiment 3: Predicted segmentation agreement values as a function of trend, framing (Endangered vs. Invasive), and spatial pattern (Distributed [purple / dark gray] vs. Clustered [orange / light gray]), derived from a mixed-effects model. (A) Model-predicted mean segmentation agreement across levels of trend, framing, and spatial pattern. Shaded areas represent 95% confidence intervals. (B) The same predicted values overlaid on observed data points (C) Marginal effects of trend on segmentation agreement across framing and spatial pattern conditions. Points represent model-estimated effects, with error bars indicating 95% confidence intervals. Full model specifications and significance tests are detailed in Tables 10 and 11

Participants gave 3.21 segmentation responses ($$SD = 2.38)$$ per stimulus on average (responses occurring within 500 ms of the previous were excluded). The mean duration of event units was 7.37 seconds ($$SD = 6.21$$). We observed a mean segmentation agreement of 0.59 ($$SD = 0.19$$). We fit a linear mixed-effects model with segmentation agreement as the outcome variable, including trend, framing, and spatial pattern, and their two- and three-way interactions as fixed effects, random intercepts, and random slopes for trend, framing, and spatial pattern per participant. The model is reported in Table 10 and Fig. 13. The hypothesized interaction between trend, framing, and spatial pattern was found to influence segmentation agreement ($$ \displaystyle \beta = 0.025, 95\% \text { CI } [0.001, 0.050], t(4823.0) = 2.071, p = .039, d = 0.129$$). The marginal effect of trend on segmentation agreement (see Table 11) was negative for invasive framing in combination with distributed change $$(\textit{AME} = -0.020$$, $$95\% \text { CI }[-0.033 \ -0.007]$$, $$p=.003)$$ and endangered framing and clustered change $$(\textit{AME} = -0.014$$, $$95\% \text { CI } [-0.027 \ -0.002]$$, $$p=.029)$$. Endangered framing with distributed change and invasive framing with clustered change did not elicit a marginal effect of trend significantly different from 0 (Fig. 14).

**Table 10:** Results of the linear mixed-effects model for segmentation agreement in Experiment 3. Fixed effects include trend [-1:1], framing [endangered vs. invasive], and spatial pattern [distributed vs. clustered], along with their two-way and three-way interactions

**Fixed Effects**							
	$\beta$	SE	95% CI		t	df	p
			Lower	Upper			
Intercept	0.591	0.008	0.575	0.606	74.789	197.7	<.001
Trend	$-$0.009	0.006	$-$0.021	0.004	$-$1.364	2067.0	.173
Framing (Invasive)	$-$0.011	0.007	$-$0.024	0.003	$-$1.540	1497.0	.124
Spatial Pattern (Clustered)	0.010	0.007	$-$0.004	0.024	1.425	697.2	.155
Trend:Framing	$-$0.011	0.009	$-$0.028	0.006	$-$1.268	4827.0	.205
Trend:Spatial Pattern	$-$0.006	0.009	$-$0.023	0.011	$-$0.670	4822.0	.503
Framing:Spatial Pattern	$-$0.003	0.010	$-$0.022	0.016	$-$0.342	4824.0	.732
Trend:Framing:Spatial Pattern	0.025	0.012	0.001	0.050	2.071	4823.0	.039

Random effects							
	Var	SD	Correlation				
Participant (Intercept)	0.006	0.081					
Participant (Trend)	0.001	0.032	0.74				
Participant (Framing)	<.001	0.009	0.03	$-$0.65			
Participant (Pattern)	<.001	0.019	$-$0.14	$-$0.78	0.98		
Residual	0.031	0.175					

Model fit							
	Marginal	Conditional					
$R^2$	0.004	0.1868					

*Note.* p values and dfs for fixed effects were calculated using Satterthwaite’s approximations. Confidence intervals were calculated using the Wald method. Model equation: segmentation agreement $\sim$ trend + framing + spatial_pattern + trend:framing + trend:spatial_pattern + framing:spatial_pattern + trend:framing:spatial_pattern + (trend + framing + spatial_pattern | participant)

**Table 11:** Marginal effects of trend on segmentation agreement depending on framing and spatial pattern in Experiment 3

Marginal effects of trend							
Framing	Pattern	AME	SE	95% CI		*z*	*p*
				Lower	Upper		
Endangered	Distributed	$-$0.009	0.007	$-$0.022	0.004	$-$1.290	.197
Endangered	Clustered	$-$0.014	0.007	$-$0.027	$-$0.002	$-$2.190	.029
Invasive	Distributed	$-$0.020	0.007	$-$0.033	$-$0.007	$-$3.001	.003
Invasive	Clustered	<.001	0.007	$-$0.013	0.013	0.013	.989

**Fig. 15:**
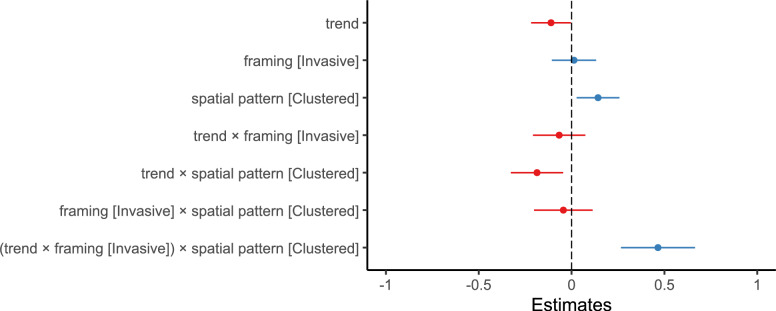
Experiment 3: Estimated effects of trend, framing (invasive), spatial pattern (clustered), and their interactions the number of segmentation responses, as derived from a mixed-effects model. The x-axis represents unstandardized parameter estimates ($\beta$), with positive values indicating increased segmentation frequency and negative values indicating reduced frequency. Error bars represent 95% confidence intervals. Estimates with confidence intervals that include zero suggest non-significant effects. Full model details, including fixed and random effects specifications, are reported in Table 12

In a post hoc analysis, we again fitted an additional linear mixed-effects model to examine the number of segmentation responses as the outcome variable, maintaining the same fixed and random effect structure as in the segmentation agreement model. The results are presented in Table 12 and Fig. 15.

Spatially clustered change elicited more frequent segmentation responses than distributed change ($\beta = 0.142, 95\% \text { CI } [0.027, 0.258], t(947.8) = 2.419, p = .016, d = 0.06$) . Additionally, the model shows a significant interaction effect between trend, framing, and spatial pattern ($\beta = 0.465, 95\% \text { CI } [0.266, 0.665], t(4710.5) = 4.569, p < .001, d = 0.197$).

**Fig. 16:**
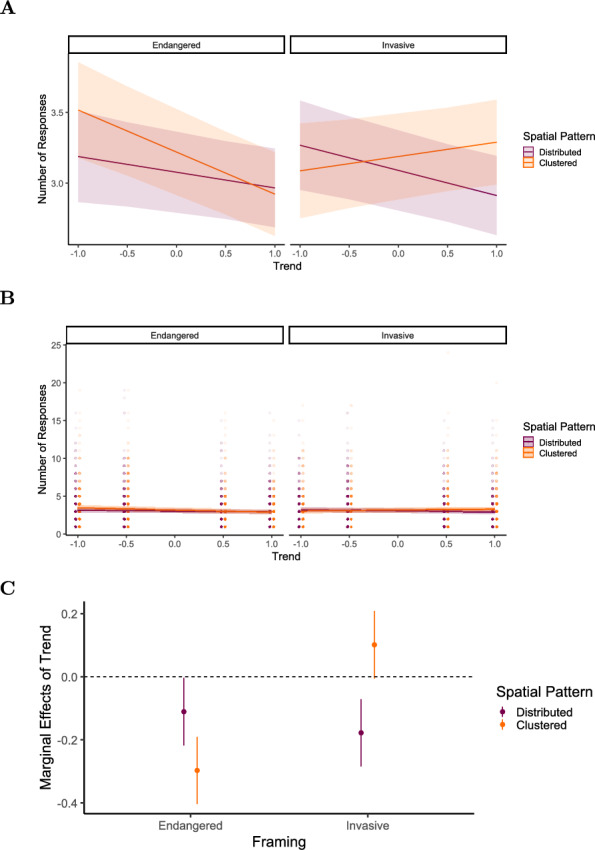
Experiment 3: Predicted number of segmentation responses as a function of trend, framing (Endangered vs. Invasive), and spatial pattern (Distributed [purple / dark gray] vs. Clustered [orange / light gray]), derived from a mixed-effects model. (A) Model-predicted mean number of segmentation responses across levels of trend, framing, and spatial pattern. Shaded areas represent 95% confidence intervals. (B) The same predicted values overlaid on observed data points (C) Marginal effects of trend on number of segmentation responses across framing and spatial pattern conditions. Points represent model-estimated effects, with error bars indicating 95% confidence intervals. Full model specifications and significance tests are detailed in Tables 12 and 13

The marginal effect of trend (Table 13) on the number of segmentation responses was negative in the endangered condition for both spatial patterns, but more pronounced for the clustered pattern $(AME = -0.305, 95\% \text { CI } [-0.412, -0.199], p <.001)$ compared to the distributed pattern $(AME = -0.118, 95\% \text { CI } [-0.225, -0.011], p =.030)$. In the invasive condition, trend had a negative effect when paired with the distributed pattern $(AME = -0.185, 95\% \text { CI } [-0.292, -0.079], p <.001)$ but a positive effect for the clustered pattern, though this effect was not statistically significant $(AME = 0.093, 95\% \text { CI } [-0.014, 0.200], p =.088)$ (see Fig. 16).

**Table 12:** Results of the linear mixed-effects model for number of segmentation responses in Experiment 3. Fixed effects include trend [-1:1], framing [endangered vs. invasive], and spatial pattern [distributed vs. clustered], along with their two-way and three-way interactions

Fixed effects							
	$\beta$	SE	95% CI		t	df	p
			Lower	Upper			
Intercept	3.077	0.144	2.796	3.358	21.442	182.0	<.001
Trend	$-$0.111	0.055	$-$0.218	$-$0.004	$-$2.031	1257.7	0.042
Framing (Invasive)	0.013	0.061	$-$0.106	0.132	0.213	610.2	0.832
Spatial Pattern (Clustered)	0.142	0.059	0.027	0.258	2.419	947.8	0.016
Trend:Framing	$-$0.067	0.072	$-$0.208	0.074	$-$0.930	4714.4	0.353
Trend:Spatial Pattern	$-$0.186	0.072	$-$0.327	$-$0.045	$-$2.589	4709.5	0.010
Framing:Spatial Pattern	$-$0.044	0.081	$-$0.202	0.114	$-$0.550	4707.8	0.582
Trend:Framing:Spatial Pattern	0.465	0.102	0.266	0.665	4.569	4710.5	<.001

Random effects							
	Var	SD	Correlation				
Participant (Intercept)	3.292	1.814					
Participant (Trend)	0.064	0.254	$-$0.640				
Participant (Framing)	0.079	0.281	$-$0.140	0.720			
Participant (Spatial Pattern)	0.037	0.194	0.620	$-$0.160	0.570		
Residual	2.115	1.454					

Model fit							
$R^2$	0.00459	0.62844					

*Note.* p values and dfs for fixed effects were calculated using Satterthwaite’s approximations. Confidence intervals were calculated using the Wald method. Model equation: number of segmentation responses $\sim$ trend + framing + spatial_pattern + trend:framing + trend:spatial_pattern + framing:spatial_pattern + trend:framing:spatial_pattern + (trend + framing + spatial_pattern | subj_id)

**Table 13:** Marginal effects of trend on number of segmentation responses depending on framing and spatial pattern in Experiment 3

Marginal effects of trend							
Framing	Spatial Pattern	AME	SE	95% CI		*z*	*p*
				Lower	Upper		
Endangered	Distributed	$-$0.118	0.055	$-$0.225	$-$0.011	$-$2.17	0.030
Endangered	Clustered	$-$0.305	0.054	$-$0.412	$-$0.198	$-$5.61	<.001
Invasive	Distributed	$-$0.185	0.054	$-$0.292	$-$0.079	$-$3.40	<.001
Invasive	Clustered	0.093	0.055	$-$0.014	0.200	1.70	0.088

### Discussion

As hypothesized, the results show a significant interaction between trend, framing, and spatial pattern in the prediction of segmentation agreement. When the change was made salient by being spatially clustered, we again observed that a negative trend in population size led to higher segmentation agreement when the species was framed as endangered. However, instead of observing a positive relationship between trend and segmentation agreement for the species framed as invasive, we find a marginal effect of trend on segmentation agreement close to zero for the clustered condition. In the distributed condition, we observe higher segmentation agreement for negative trends in both framing conditions. Higher segmentation agreement for the clustered than the distributed condition indicates that the more subtle manipulation of change salience was successful. As in Experiment 2, exploratory analyses showed that segmentation frequency increased in the same conditions as segmentation agreement: when framing and trend were congruent in the high-salience clustered spatial pattern.

Overall, these results, in principle, replicate the interaction found in experiment 2, but the pattern of the interaction is not replicated exactly: We find a neutral instead of a positive relationship between trend and segmentation agreement in the clustered and invasive condition. Due to the spatial pattern manipulation, the range of trends depicted in the maps was restricted to a subset of regions, making it more difficult to perceive. Perhaps this dampened the effect of the trend manipulation on segmentation agreement. The fact that this weaker manipulation still led to an overall interaction with framing and salience supports the robustness of the underlying effect.

The mean segmentation agreement of 0.59 is similar to the mean segmentation agreement recorded in Experiment 2. Notably, we also observe comparable variances in the random intercepts fit per the subject and residual variance in experiments 2 and 3 (see Tables 6 and 10), indicating that those are stable estimates across participant groups and stimuli.

## General Discussion

We conducted three experiments to test if findings from event segmentation research generalize to data visualizations in the form of dynamic thematic maps and which factors influence the shared processing of such stimuli.

### Meaningful segmentation agreement on dynamic maps

The first results demonstrate that people agree on the temporal placement of event boundaries for abstract data depictions on maps above chance level and to a similar degree as found in prior studies for moving geometric shapes (Zacks, 2004), commercials, and everyday scenes (Sasmita and Swallow, 2022). This is noteworthy due to the fundamental differences between the map stimuli and the type of material for which segmentation is well studied: The dynamic maps do not provide prior event schemata, actors whose intentions could be inferred or even objects whose movement features could elicit event boundaries. These findings, therefore, show that event segmentation is a basal process in the perception of dynamic stimuli that does not rely on direct intentions driving the observed changes or movement of objects as triggers. We found no between-group difference in the segmentation of maps with different timeline units. An exploratory analysis revealed that participants agreed to a higher degree when segmenting maps showing an increasing rather than decreasing trend in the depicted data.

### Conceptual and perceptual features impact agreement

Building on these findings, the two main experiments more closely examined how the depicted data trend interacts with the conceptual and perceptual features of the maps in influencing segmentation agreement. Maps showing how the population densities of (fictional) insect species developed on (fictional) islands were used as stimuli. Both in experiments 2 and 3, the conceptual interpretation or expectation regarding the depicted trend was manipulated by framing the insect species as either endangered or invasive prior to and during the presentation of the maps. Additionally, the perceptual salience of the direction of the depicted trend was manipulated to create a high- and a low-salience condition. In Experiment 2, perceptual salience was operationalized through the color scales used to depict the population sizes. While saturation-based color scales make it easy to judge the direction of the trend (high salience), hue-based color scales make it more difficult (low salience). In Experiment 3, the salience of the direction of change was manipulated through the spatial pattern of the change. Only a subset of the maps’ regions showed the trend, and those were either clustered together (high salience) or distributed across the map (low salience). We hypothesized higher segmentation agreement when the depicted trend matched the expectation elicited by the framing, but only in the high-salience condition. Confirming this hypothesis, the results from Experiment 2 showed a significant interaction between the depicted trend, the provided framing, and the salience of the direction of change in the prediction of segmentation agreement in the predicted pattern. Experiment 3 showed a significant interaction between the depicted trend, the provided framing, and the salience of the direction of change in the prediction of segmentation agreement as well. However, negative trends matching the expectation elicited by the framing as endangered led to higher agreement in both salience conditions. For invasive framing, the trend did not influence agreement in the high-salience condition. In the low-salience condition, for which no influence was hypothesized, positive trends led to lower agreement than negative trends. This was surprising, as the previous experiments showed higher agreement for positive trends in general (Experiment 1, see Fig. 6) and higher agreement for positive trends when those positive trends were expected due to species being described as invasive, specifically (Experiment 2, see Fig. 9). A likely explanation for this deviation for the low-salience condition of Experiment 3 (Fig. 16) is that the influence of trend is subdued in two ways: Once by the principal change made in comparison with Experiment 2, that the trend is only shown in a subset of regions. Consequently, when comparing results between experiments, it is important to keep in mind that across the whole map, the strongest trend in Experiment 3 corresponds to a moderate trend in Experiment 2. Second, in the low-salience condition, these change-depicting regions are spread across the map, making the trend harder to perceive. Therefore, we interpret the unexpected result pattern in the low-salience condition as the combined result of reduced perceptual clarity and attenuated overall trend strength, which may have obscured any advantage for positive or expectation-congruent trends that was observed in the earlier experiments.

Across experiments 2 and 3, exploratory analyses of the frequency of segmentation responses revealed similar patterns. Participants segmented maps for which the framing and the trend matched more finely if the direction of change was salient.

These results show that segmentation is modulated both by bottom-up perceptual features of the stimuli and by the top-down influences such as expectations and prior knowledge of the view, which fit well into prior research findings (Zacks, 2004, 2020; Newberry et al., 2021). The maps’ underlying trend is made more difficult to discern by noise added to the depicted data, which could have led to an overall ambiguous impression for the participants. The framing manipulation might have decreased that perceived ambiguity when the framing matched the depicted trend, easing and converging segmentation. This effect has already been shown for ambiguous texts (Newberry and Bailey, 2019): Participants segmenting ambiguous text with a title agreed more strongly in their segmentation than those who segmented the same texts without a title. One could also interpret the influence of framing as a confirmation bias in identifying event boundaries.

### Reducing ambiguity promotes meaningful prediction error dynamics

These findings align with prediction-based theories of event segmentation, which describe event models as internal representations that generate expectations about upcoming perceptual input (Kurby and Zacks, 2008). When incoming information violates these expectations, a transient increase in prediction error triggers an update to the event model, resulting in the perception of an event boundary (Zacks and Swallow, 2007). Supporting this, Kumar et al. (2023) show that Bayesian surprise—a measure of abrupt change in predictive belief states—is a more robust predictor of segmentation than raw surprisal or entropy. Similarly, transient spikes in prediction error, rather than sustained high uncertainty, have been shown to better predict boundaries in computational models (Reynolds et al., 2007) and are associated with physiological markers of boundary detection such as pupil-linked arousal (Clewett et al., 2020).

In the case of the dynamic map stimuli, segmentation may depend on the ability to construct stable event models in the first place. Participants may struggle to generate predictions when low change direction salience or incongruent framing makes the underlying trend difficult to interpret. In such incongruent conditions, the mismatch between framing and depicted trends likely hinders the formation of a stable event model, raising the baseline of prediction error and making spikes less pronounced. In other words, the incongruence may result in sustained but uninformative prediction errors, decreasing segmentation frequency and agreement. This is consistent with the lower number of segmentation responses observed for incongruent maps, suggesting not only more variable timing of boundary placement but also an overall reduction in the perception of distinct boundaries. In contrast, when framing supports the perceptual trend, e.g., when an endangered species’ population declines as expected, the reduction in ambiguity may enable a more stable event model to form, increasing the likelihood of transient prediction errors when meaningful changes occur. In summary, predictions must be possible for prediction errors to serve their role in event model updating. This is particularly relevant in abstract stimuli such as dynamic maps, for which event schemata are less available. This is further reinforced by increased segmentation agreement in conditions where framing matched trend direction, suggesting that shared expectations facilitated event model construction across participants.

### High inter-individual variance in segmentation agreement

The estimated parameters of the confirmed three-way-interaction effects of trend, framing, and salience on agreement ($\beta _{T:F:C}$ $=$ 0.046, $\beta _{T:F:P}$ $=$ 0.024) are not large. They have to be interpreted in the context of the discussed noise present in the stimuli, which partially masks the depicted trend and thereby limits its influence on segmentation. The stimuli used in experiment 3 exhibited a smaller signal-to-noise ratio than those used in experiment 2 due to the spatial pattern manipulation. The relatively smaller influence of the trend (the signal in that case), its framing, and salience in experiment 3 could be attributable to this higher noise.

Large inter-individual differences in segmentation agreement, as reflected by variance in participant intercepts ($SD_{p1}$ $=$ 0.097, $SD_{p2}$ $=$ 0.117) are in line with results for other classes of stimuli (Zacks, 2020). As age is a factor that has been shown to influence segmentation (Sargent et al., 2013; Zacks, 2020), the large age span in our participant groups might have contributed to the high variances found. Sava-Segal et al. (2023) studied individual differences in neural correlates of event segmentation, revealing higher variability in cortical regions linked to higher-order associations than those linked to sensory processing. In the context of our experiments, different levels of prior knowledge of and interest in topics of biodiversity might elicit different associations with probable causes and consequences of changes in insect populations and thereby contribute to high variability in segmentation.

Another factor potentially contributing to high variances is the online collection of data. The conditions in which the participants completed the experiment were not as controlled as in a laboratory setting, and different levels of distraction might impact segmentation. At the same time, this experimental setting might more closely mimic how people consume data maps similar to those used in the experiment, increasing ecological validity.

All in all, the found effects are not large, but that is to be expected in such a high-variance task performed on complex stimuli. Moreover, the key result of an interaction between trend, framing, and salience found in experiment 2 was partly replicated in experiment 3 with a different salience manipulation. This suggests that these small but consistent effects are robust across varying conditions.

From a practical viewpoint, the found effects are of interest because they provide options for improving the design of dynamic maps regardless of their viewers’ age or watching conditions.

This is especially relevant as large spatiotemporal datasets become increasingly available (Stewart et al., 2015; Lloyd et al., 2019), and open software solutions reduce the barriers to creating animated data maps (Fish, 2015; Usher, 2020); the prevalence of these maps in online media grows. It is important to also note that the necessary information aggregation might hide the political biases of the map-makers, oversimplify issues, and ignore those not represented in the depicted data (Usher, 2020). These epistemological challenges highlight the importance of investigating the basic perceptual and cognitive processes involved in reading these maps as a necessary step toward understanding the interplay between data, map-makers, and map users ((Koláčny, 1969).

### Constraints on Generality (COG)

To discuss the constraints on the generality of our findings as proposed by Simons et al. (2017), we will consider the experiments’ participant samples, the studied stimuli, and the potential constraints of the methodology.

We recruited participants online, requiring self-reported fluency in English and normal or corrected-to-normal vision. The broad inclusion criteria helped to obtain data from participants of a wide range of ages and locations across the world. As discussed above, the samples provided substantial variance in the outcome variables. We would, therefore, expect that the findings generalize to the general population of people who might come across dynamic maps online. However, online recruitment may still skew the sample toward populations with higher digital literacy and familiarity with dynamic visualizations.

The stimuli used across the three experiments showed fictional areas to avoid familiarity influences. We showed that participants’ expectations—elicited by the framing of species as endangered or invasive—shape the segmentation process. Whether this finding generalizes to expectations held by participants due to their general knowledge of a geographic area or the thematic data shown on a map is a question for future research.

Concerning the generalizability of the findings to different dynamic maps, we also have to consider their complexity. As a first step in investigating event segmentation of thematic dynamic maps, we chose to display very simple data patterns that overall increased or decreased monotonically and linearly. However, many data patterns produced by the real world are more complicated. We witnessed the importance of understanding exponential growth during the COVID-19 pandemic (Komarova et al., 2020). In the context of the climate crisis, communication regarding tipping points and how crossing them may accelerate changes and damages in the climate system is an important and challenging task (Nadeau et al., 2024). A clear future direction is therefore to investigate the influence of map data complexity, specifically motion dynamics and spatial autocorrelation of real-world processes, on segmentation agreement. A focus on the statistical properties of natural data would allow the development of a theory linking these dynamics to event models and the processes of meaning-making in thematic map interpretation based on the information available in such displays.

Event segmentation has been shown to lay the basis for organizing memory processes (Radvansky and Zacks, 2017): Boundaries are recalled with higher degrees of detail than event middles. Cueing event boundaries during observation can also improve memory (Gold et al., 2017). More normative segmentation is associated with improved memory (Bailey et al., 2013), an effect independent of event knowledge (Sargent et al., 2013). Future research will have to examine if this established relationship between segmentation and memory generalizes to dynamic map stimuli as well.

Furthermore, future research might elaborate more on the motives and ways of use of map readers and how they impact segmentation outside a controlled experimental set-up. For example, a person eager to extract information from a dynamic map will watch it not once, as in the current experimental design, but several times. Martin and Tversky (2013) showed that watching clips of moving geometric shapes five times compared to one time changes how strongly participants conceptualize what they observe but not the way they segment it. This raises the question of whether repeated presentation may accentuate the influence of a conceptual framing manipulation such as ours on segmentation. Additional aspects to consider going forward in the real-world use of dynamic maps are contextualization, such as embedding in an online news article, the perceived trustworthiness of the data and map source, the polarity of the depicted topics, and the viewer’s attitudes and beliefs toward those topics.

Overall, while our findings offer first insights into event segmentation in dynamic thematic maps, their generalizability remains contingent on stimulus complexity, cognitive biases, contextual embedding, and real-world user behaviors. Future studies should explore how these factors interact to shape segmentation processes across diverse settings and populations.

### Conclusion

To our knowledge, this research is the first to apply an event segmentation theory paradigm to data visualizations.

The results of the three experiments allow two inferences. First, people exhibit similar levels of agreement when segmenting dynamic maps as they do for naturalistic scenes. Second, segmentation is influenced both by conceptual factors such as expectations and schema and by perceptual features of the maps like the salience of directional changes. Our findings suggest that event segmentation processes apply not only to dynamic scenes involving agents and actions but also to abstract, symbolic representations–provided that such representations support coherent interpretation through perceptual and conceptual cues. This reinforces the view that event segmentation is a fundamental aspect of human perception and sense-making, irrespective of the nature of the continuous experience (Radvansky and Zacks, 2017).

From an applied perspective, the influence of the color scale used underscores the importance of congruent transitions between values in dynamic maps, with segmentation agreement serving as a novel quantitative cognitive measure in this context (Battersby and Goldsberry, 2010). More interestingly, the pattern that people agree more when they see what they expect to see in the maps supports the notion that data cannot always speak for itself. Particularly when conveying unexpected dynamics, integrating maps with verbal information and highlighting key observations can facilitate shared understanding and subsequent dialogue about the findings. However, this approach to data storytelling may also enhance the processing of available information, potentially introducing biases (Usher, 2020).

Overall, this research demonstrates the inherent complexity of effectively communicating complex data through dynamic maps and shows how empirical work grounded in cognitive theories can contribute to understanding and improving this process.

## Data Availability

Data, stimuli, and analysis code for all three experiments can be found here: https://osf.io/v9n3m/overview The experiments were presented using JsPsych (De Leeuw, 2015). Analyses were carried out with R R Core Team (2021), using the *lme4* (Bates et al., 2015) and the *marginaleffects* packages (Arel-Bundock et al., 2024).

## ﻿Appendix A Instruction Comprehension Check - Pilot Experiment

Over which time span has the data you will be reviewing been collected?

- **10 Days**
- 10 Weeks
- 10 Months
- **10 Years**

The correct response depended on the condition.

Which key are you supposed to press to indicate that you perceive that one meaningful event has ended and another meaningful event has begun?

- Enter
- **Spacebar**
- Arrow Up
- Arrow Down

## Appendix B Instruction Comprehension Check—Main Experiments

What aspect of insect populations will be shown on the maps?

- Insect behavior
- Habitat types
- **Population size**
- 10 Years

Which key are you supposed to press to indicate that you perceive that one meaningful event has ended and another meaningful event has begun?

- Enter
- **Spacebar**
- Arrow Up
- Arrow Down

What makes a species invasive?

- They have natural predators in their new environment
- They are native to a certain place
- They reproduce slowly and have a stable population
- **They lack natural predators in their new environment**

What is an endangered species?

- Species that are not native to a certain place
- **Species that are at risk of disappearing forever**
- Species that are causing harm to the environment
- Species that reproduce and spread rapidly

## ﻿Appendix C Experiment 2: Model Formula

$$\begin{aligned} \text{agreement}_{i}&\sim N \left( \mu , \sigma ^2 \right) \\ \mu&=\alpha _{j[i]}\ + \\&\quad \beta _{1j[i]}(\text{trend})\ + \\&\quad \beta _{2j[i]}(\text{framing}_{\text{Invasive}})\ + \\&\quad \beta _{3j[i]}(\text{color\_scale}_{\text{Saturation}})\ + \\&\quad \beta _{4}(\text{framing}_{\text{Invasive}} \times \text{trend})\ + \\&\quad \beta _{5}(\text{color\_scale}_{\text{Saturation}} \times \text{trend})\ + \\&\quad \beta _{6}(\text{color\_scale}_{\text{Saturation}} \times \text{framing}_{\text{Invasive}})\ + \\&\quad \beta _{7}(\text{color\_scale}_{\text{Saturation}} \times \text{framing}_{\text{Invasive}} \times \text{trend}) \\ \left( \begin{array}{c} \begin{aligned} & \alpha _{j} \\ & \beta _{1j} \\ & \beta _{2j} \\ & \beta _{3j} \end{aligned} \end{array} \right)&\sim N \left( \left( \begin{array}{c} \begin{aligned} & \mu _{\alpha _{j}} \\ & \mu _{\beta _{1j}} \\ & \mu _{\beta _{2j}} \\ & \mu _{\beta _{3j}} \end{aligned} \end{array} \right) , \left( \begin{array}{cccc} \sigma ^2_{\alpha _{j}} & \rho _{\alpha _{j}\beta _{1j}} & \rho _{\alpha _{j}\beta _{2j}} & \rho _{\alpha _{j}\beta _{3j}} \\ \rho _{\beta _{1j}\alpha _{j}} & \sigma ^2_{\beta _{1j}} & \rho _{\beta _{1j}\beta _{2j}} & \rho _{\beta _{1j}\beta _{3j}} \\ \rho _{\beta _{2j}\alpha _{j}} & \rho _{\beta _{2j}\beta _{1j}} & \sigma ^2_{\beta _{2j}} & \rho _{\beta _{2j}\beta _{3j}} \\ \rho _{\beta _{3j}\alpha _{j}} & \rho _{\beta _{3j}\beta _{1j}} & \rho _{\beta _{3j}\beta _{2j}} & \sigma ^2_{\beta _{3j}} \end{array} \right) \right) \\ \text {for subj\_id } j&= 1, \dots \text {, J} \end{aligned}$$

## ﻿Appendix D Experiment 3: Model Formula

$$\begin{aligned} \text{agreement}_{i}&\sim N \left( \mu , \sigma ^2 \right) \\ \mu&=\alpha _{j[i]}\ + \\&\quad \beta _{1j[i]}(\text{trend})\ + \\&\quad \beta _{2j[i]}(\text{framing}_{\text{Invasive}})\ + \\&\quad \beta _{3j[i]}(\text{spatial\_pattern}_{\text{Clustered}})\ + \\&\quad \beta _{4}(\text{framing}_{\text{Invasive}} \times \text{trend})\ + \\&\quad \beta _{5}(\text{spatial\_pattern}_{\text{Clustered}} \times \text{trend})\ + \\&\quad \beta _{6}(\text{framing}_{\text{Invasive}} \times \text{spatial\_pattern}_{\text{Clustered}})\ + \\&\quad \beta _{7}(\text{framing}_{\text{Invasive}} \times \text{spatial\_pattern}_{\text{Clustered}} \times \text{trend}) \\ \left( \begin{array}{c} \begin{aligned} & \alpha _{j} \\ & \beta _{1j} \\ & \beta _{2j} \\ & \beta _{3j} \end{aligned} \end{array} \right)&\sim N \left( \left( \begin{array}{c} \begin{aligned} & \mu _{\alpha _{j}} \\ & \mu _{\beta _{1j}} \\ & \mu _{\beta _{2j}} \\ & \mu _{\beta _{3j}} \end{aligned} \end{array} \right) , \left( \begin{array}{cccc} \sigma ^2_{\alpha _{j}} & \rho _{\alpha _{j}\beta _{1j}} & \rho _{\alpha _{j}\beta _{2j}} & \rho _{\alpha _{j}\beta _{3j}} \\ \rho _{\beta _{1j}\alpha _{j}} & \sigma ^2_{\beta _{1j}} & \rho _{\beta _{1j}\beta _{2j}} & \rho _{\beta _{1j}\beta _{3j}} \\ \rho _{\beta _{2j}\alpha _{j}} & \rho _{\beta _{2j}\beta _{1j}} & \sigma ^2_{\beta _{2j}} & \rho _{\beta _{2j}\beta _{3j}} \\ \rho _{\beta _{3j}\alpha _{j}} & \rho _{\beta _{3j}\beta _{1j}} & \rho _{\beta _{3j}\beta _{2j}} & \sigma ^2_{\beta _{3j}} \end{array} \right) \right) \\ \text {for subj\_id} j&= 1, \dots \text {,J} \end{aligned}$$
